# Supplementary material for: Local nutrient addition drives plant diversity losses but not biotic homogenization in global grasslands
Source: Nat Commun. 2025 May 27;16:4903. doi: 10.1038/s41467-025-59166-7 (PMC12116774; doi:10.1038/s41467-025-59166-7)
Supplement: Supplementary file 1 — Supplementary Information [file 41467_2025_59166_MOESM1_ESM.pdf]

Supplementary Materials for

Local nutrient addition drives plant diversity losses but not biotic homogenization in global grasslands

Qingqing Chen[1,2*], Shane A. Blowes[1,3], W. Stanley Harpole[1,4,5], Emma Ladouceur[1,3,6–8], Elizabeth T. Borer[9], Andrew MacDougall[10], Jason P. Martina[11], Jonathan D. Bakker[12], Pedro M. Tognetti[13], Eric W. Seabloom[9], Pedro Daleo[14], Sally Power[15], Christiane Roscher[1,4], Peter B. Adler[16], Ian Donohue[17], George Wheeler[18], Carly Stevens[19], G.F. Ciska Veen[20], Anita C. Risch[21], Glenda M. Wardle[22], Yann Hautier[23], Catalina Estrada[24], Erika Hersch-Green[25], Yujie Niu[26], Pablo L. Peri[27], Anu Eskelinen[1,4,28],Daniel S. Gruner[29], Harry Olde Venterink[30], Carla D'Antonio[31],Marc W. Cadotte[32], Sylvia Haider[1,33], Nico Eisenhauer[1,34], Jane Catford[35,36], Risto Virtanen[28], John W. Morgan[37], Michelle Tedder[38], Sumanta Bagchi[39], Maria C. Caldeira[40], Miguel N. Bugalho[41], Johannes M. H. Knops[42], Chris R. Dickman[43], Nicole Hagenah[44], Anke Jentsch[26], Petr Macek[45], Brooke B. Osborne[46], Lauri Laanisto[47], Jonathan M. Chase[1,3]
Corresponding author: chqq365@gmail.com

The PDF file includes:

Figs. S1 to S10

Tables S1 to S8

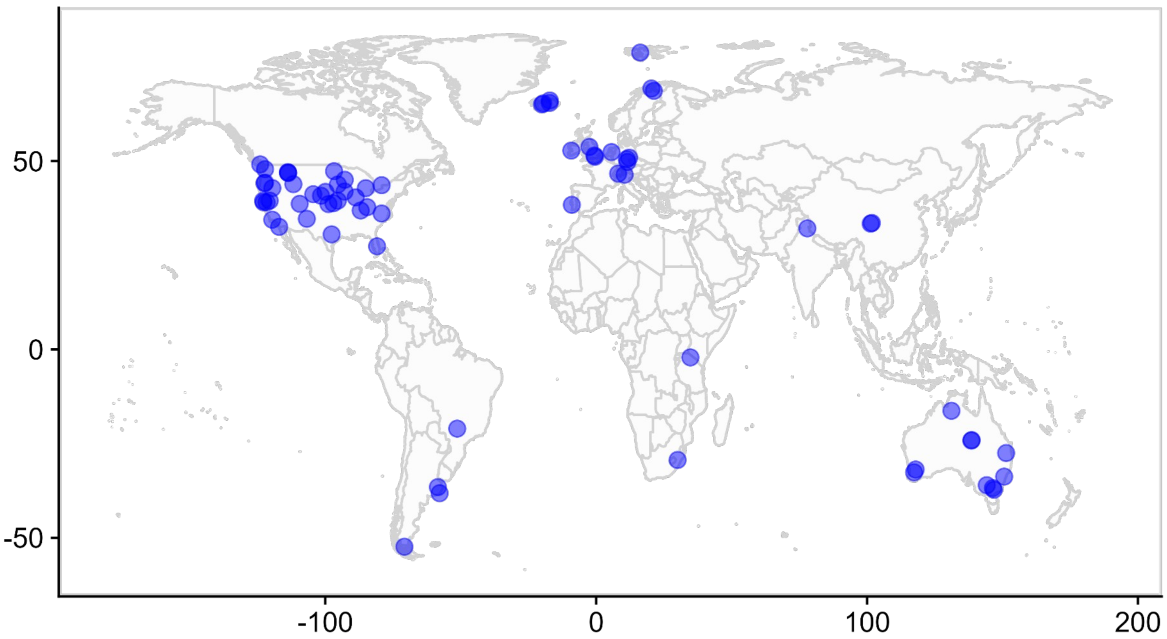

Fig. S1.

Geolocation of the 72 sites used in this analysis. See table S1 for more detail about site information such as habitat type, experimental years. Source data are provided as a Source Data file. Base map from the R package "maps", accessed via ggplot2::map_data("world"). Source for base map: Natural Earth (https://www.naturalearthdata.com).

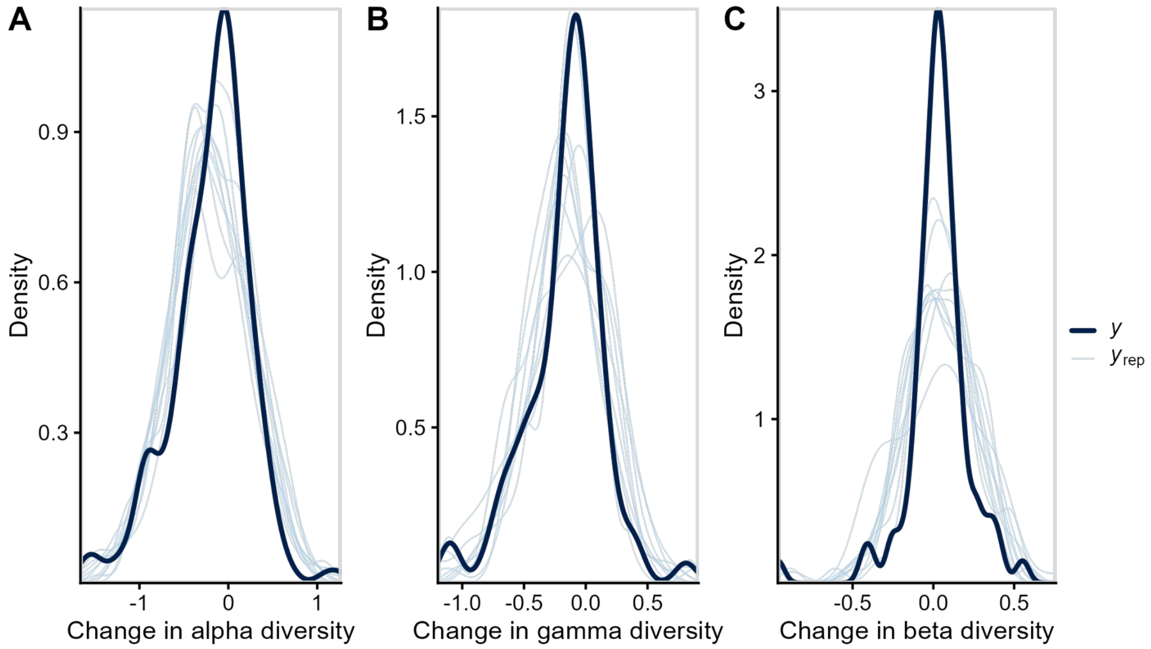

Fig. S2.

Predicted values (the thin lines) and observed values (the thick lines) for change in alpha, gamma, and beta diversity (Δα, Δγ, and Δβ) under nutrient addition. (A) Δα, (B) Δγ, (C) Δβ. Model predicted values reasonably well. See Table S3 for more detail about model fitting.

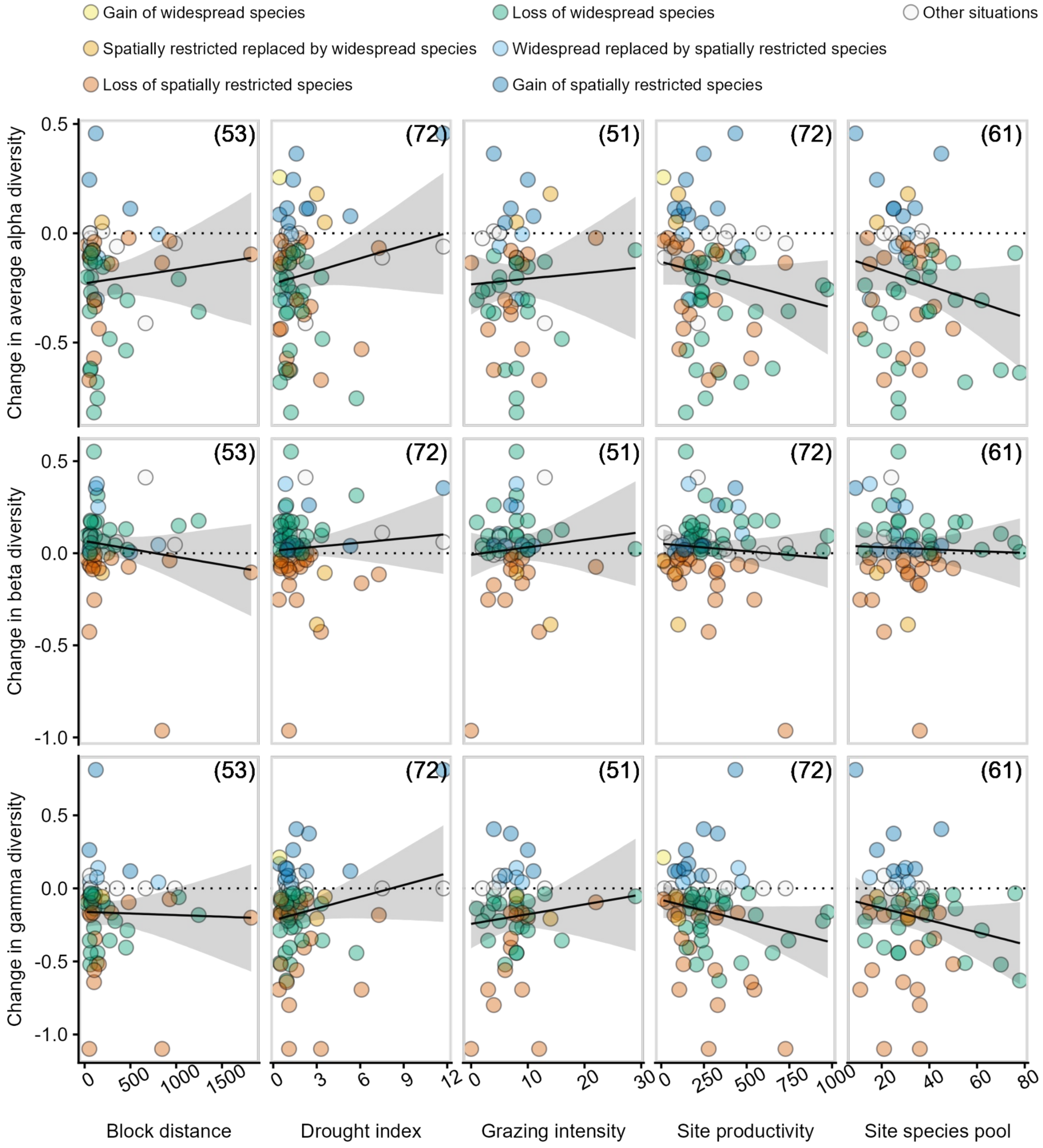

Fig. S3.

Bivariate relationship between change in average alpha, gamma, and beta diversity ($\overline{\Delta\alpha}$, Δγ, and Δβ) 4 years after treatment began and site covariates. Unit for block distance is meter, unit for site productivity is g m^{-2}. Solid black lines represent linear regression lines, grey areas are 95% credible bands. None of the relationships are statistically significant. Numbers in the parentheses are the number of sites. Other scenarios: $\overline{\Delta\alpha}$ = 0, Δγ = 0, or Δβ = 0. When a site has these situations, it was not counted into any of the six scenarios in the framework. Site sier.us was excluded for the relationship between block distance and change in diversity because blocks were arranged very far away from each other (12538.09 m), $\overline{\Delta\alpha}$, Δγ, and Δβ was 0.364, 0.405, and 0.041, respectively. Source data are provided as a Source Data file.

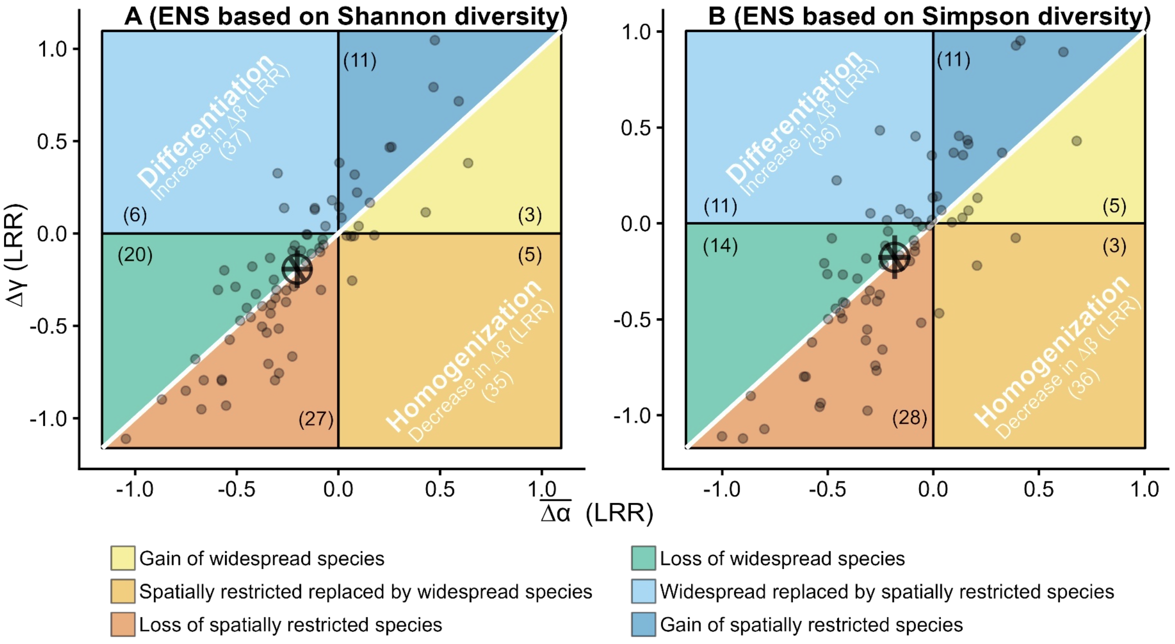

Fig. S4.

Changes in average α, γ, and β diversity ($\overline{\Delta\alpha}$, Δγ, and Δβ) with nutrient addition using effective number of species. (A) based on Shannon diversity, (B) based on Simpson diversity. ENS: effective number of species; LRR: log response ratio. The white 1:1 diagonal line indicates no effects of nutrient addition on β diversity. Numbers in the parentheses are the number of sites. When a site has $\overline{\Delta\alpha}$ = 0, Δγ = 0, or Δβ = 0, it was not counted into any of the six scenarios in the framework. The small points represent site-level $\overline{\Delta\alpha}$, Δγ, and Δβ at 72 sites. The large open point and error bars are the estimated mean and 95% credible intervals for $\overline{\Delta\alpha}$, Δγ, and Δβ across all sites. See Table S3 for model fit and estimated overall means and 95% credible intervals for $\overline{\Delta\alpha}$, Δγ, and Δβ. Source data are provided as a Source Data file.

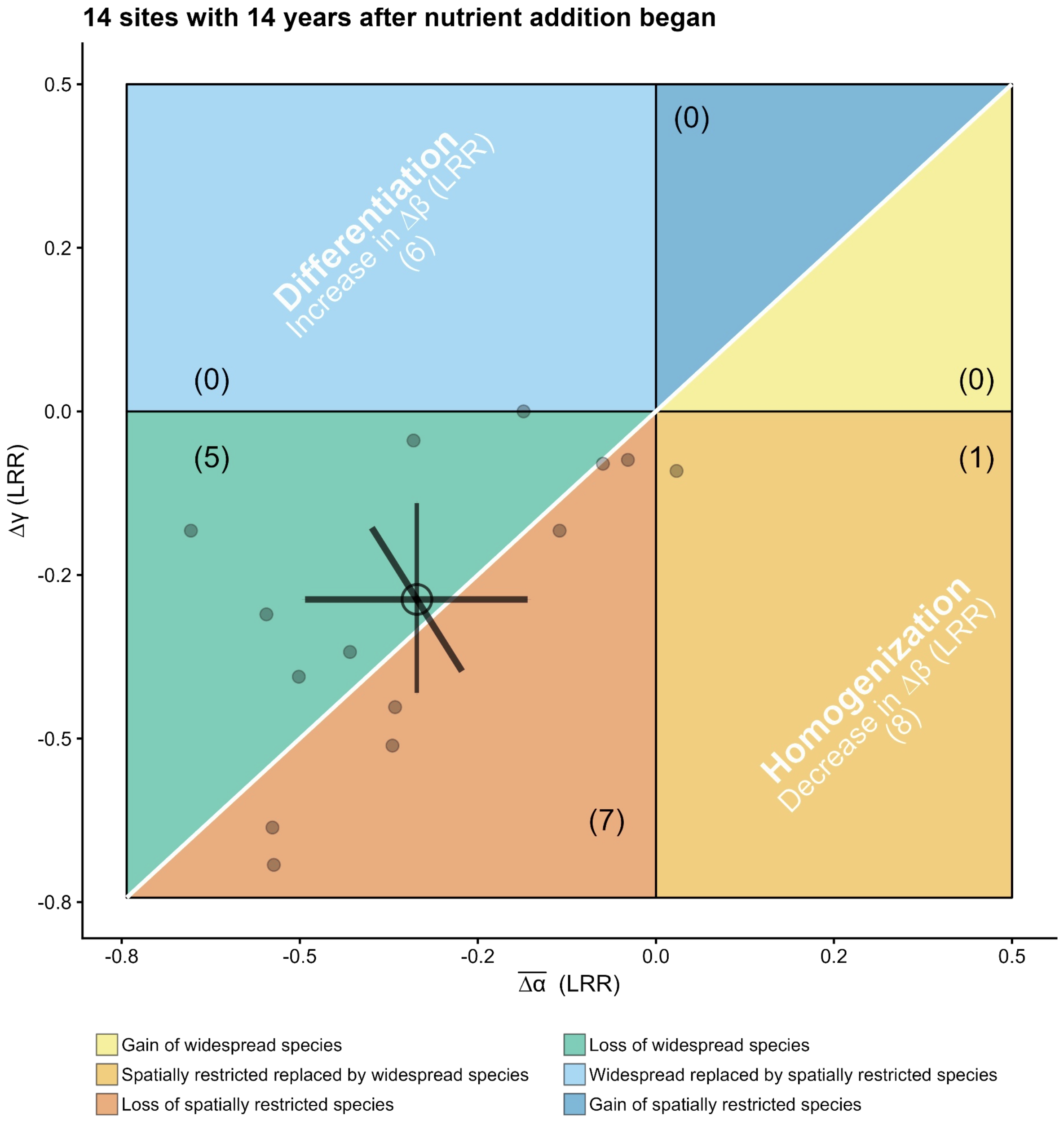

Fig. S5.
Changes in average α, γ, and β diversity ($\overline{\Delta\alpha}$, Δγ, and Δβ) with nutrient addition 14 years after treatment began. LRR: log response ratio. The white 1:1 diagonal line indicates no effects of nutrient addition on β diversity. Numbers in the parentheses are the number of sites. When a site has $\overline{\Delta\alpha}$ = 0, Δγ = 0, or Δβ = 0, it was not counted into any of the six scenarios. The small points represent site-level $\overline{\Delta\alpha}$, Δγ, and Δβ. The large open point and error bars are the estimated mean and 95% credible intervals for $\overline{\Delta\alpha}$, Δγ, and Δβ across all sites. Source data are provided as a Source Data file.

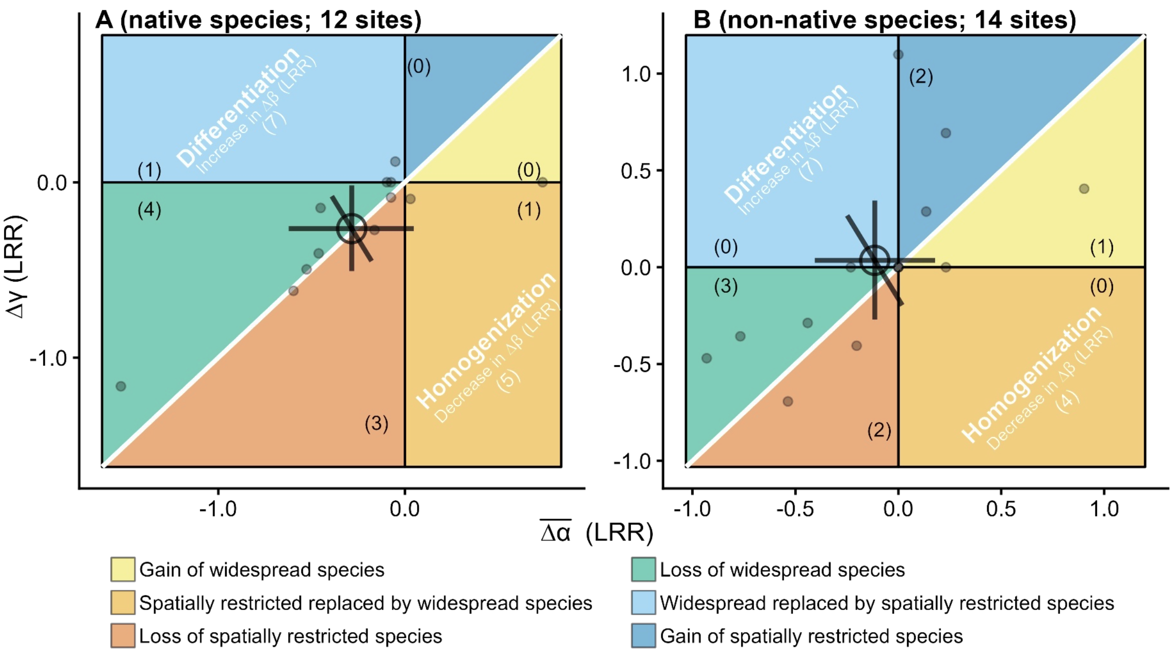

Fig. S6.

Changes in average α, γ, and β diversity ($\overline{\Delta\alpha}$, Δγ, and Δβ) with nutrient addition for native and non-native species groups 14 years after treatment began. (A) native and (B) non-native species. LRR: log response ratio. The white 1:1 diagonal line indicates no effects of nutrient addition on β diversity. Numbers in the parentheses are the number of sites. When a site has $\overline{\Delta\alpha}$ = 0, Δγ = 0, or Δβ = 0, it was not counted into any of the six scenarios. The small points represent site-level $\overline{\Delta\alpha}$, Δγ, and Δβ. The large open point and error bars are the estimated mean and 95% credible intervals for $\overline{\Delta\alpha}$, Δγ, and Δβ across all sites. Source data are provided as a Source Data file.

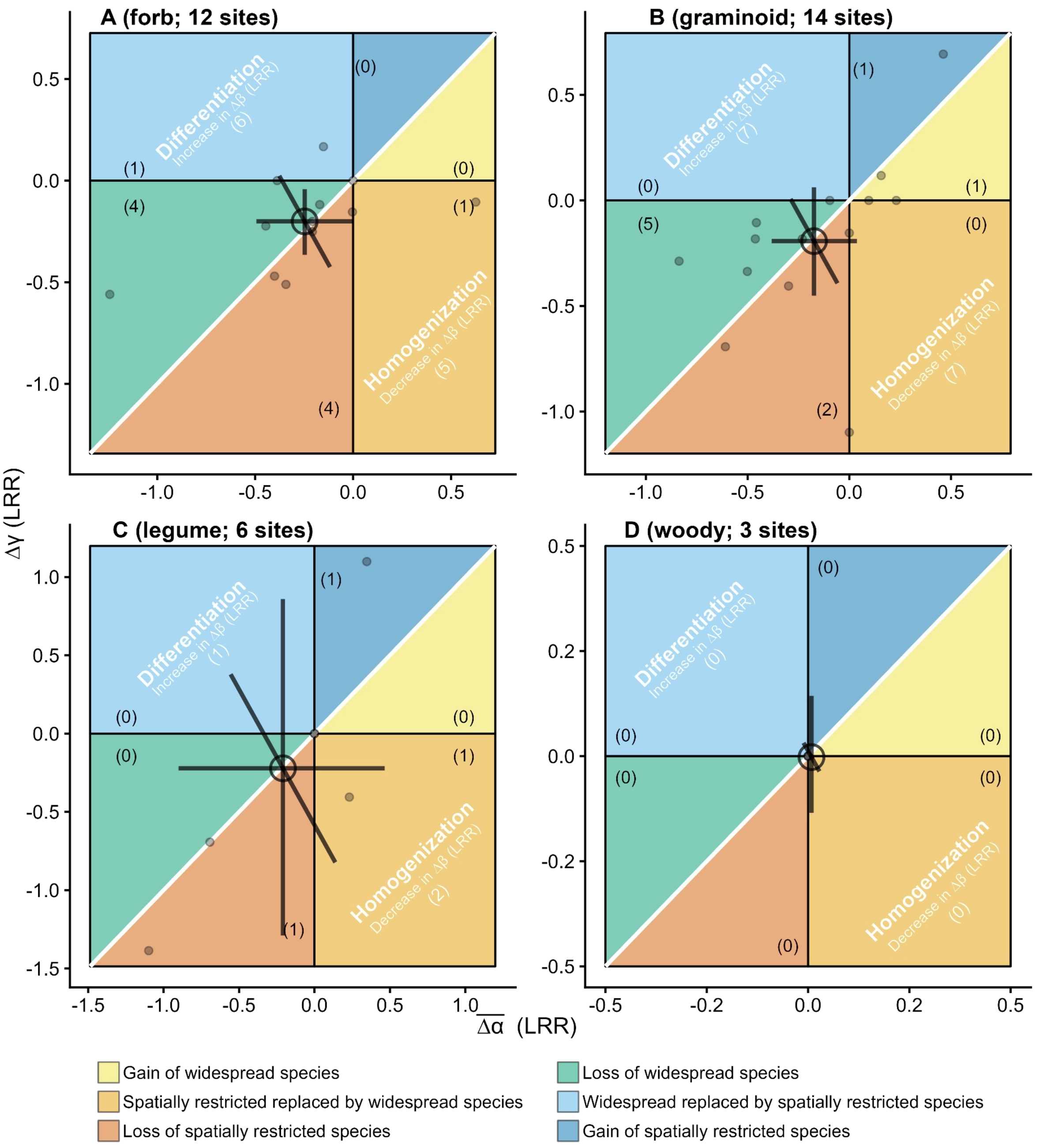

Fig. S7.

Changes in average α, γ, and β diversity ($\overline{\Delta\alpha}$, Δγ, and Δβ) with nutrient addition for different functional species groups 14 years after treatment began. (A) forb, (B) graminoid, (C) legume, and (D) woody species. LRR: log response ratio. The white 1:1 diagonal line indicates no effects of nutrient addition on β diversity. Numbers in the parentheses are the number of sites. When a site has $\overline{\Delta\alpha}$ = 0, Δγ = 0, or Δβ = 0, it was not counted into any of the six scenarios. The small points represent site-level $\overline{\Delta\alpha}$, Δγ, and Δβ. The large open point and error bars are the estimated mean and 95% credible intervals for $\overline{\Delta\alpha}$, Δγ, and Δβ across all sites. Source data are provided as a Source Data file.

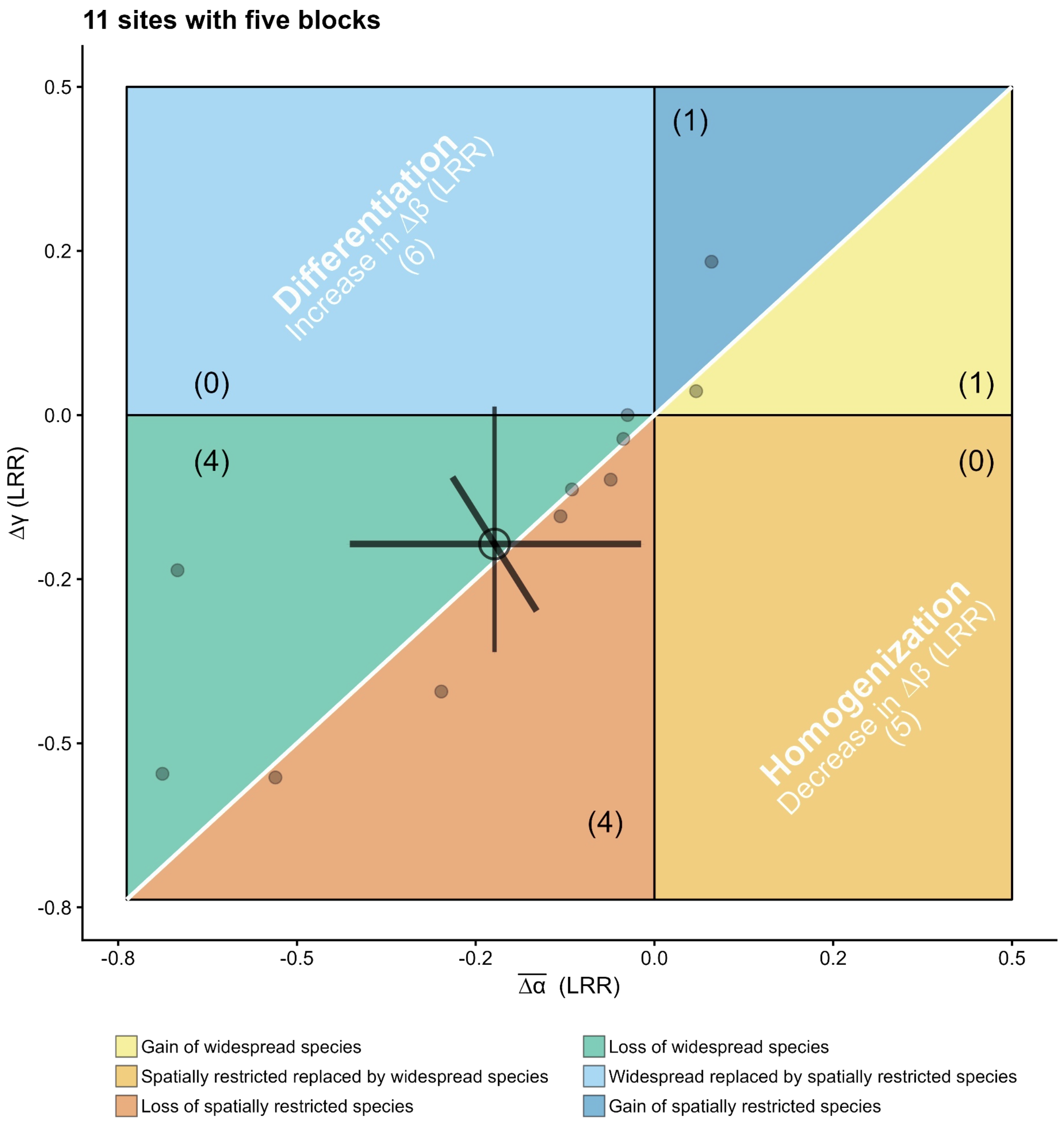

Fig. S8.

Changes in average α, γ, and β diversity ($\overline{\Delta\alpha}$, Δγ, and Δβ) with nutrient addition for sites with five blocks. LRR: log response ratio. The white 1:1 diagonal line indicates no effects of nutrient addition on β diversity. Numbers in the parentheses are the number of sites. When a site has $\overline{\Delta\alpha} = 0$, $\Delta\gamma = 0$, or $\Delta\beta = 0$, it was not counted into any of the six scenarios. The small points represent site-level $\overline{\Delta\alpha}$, Δγ, and Δβ. The large open point and error bars are the estimated mean and 95% credible intervals for $\overline{\Delta\alpha}$, Δγ, and Δβ across all sites. Source data are provided as a Source Data file.

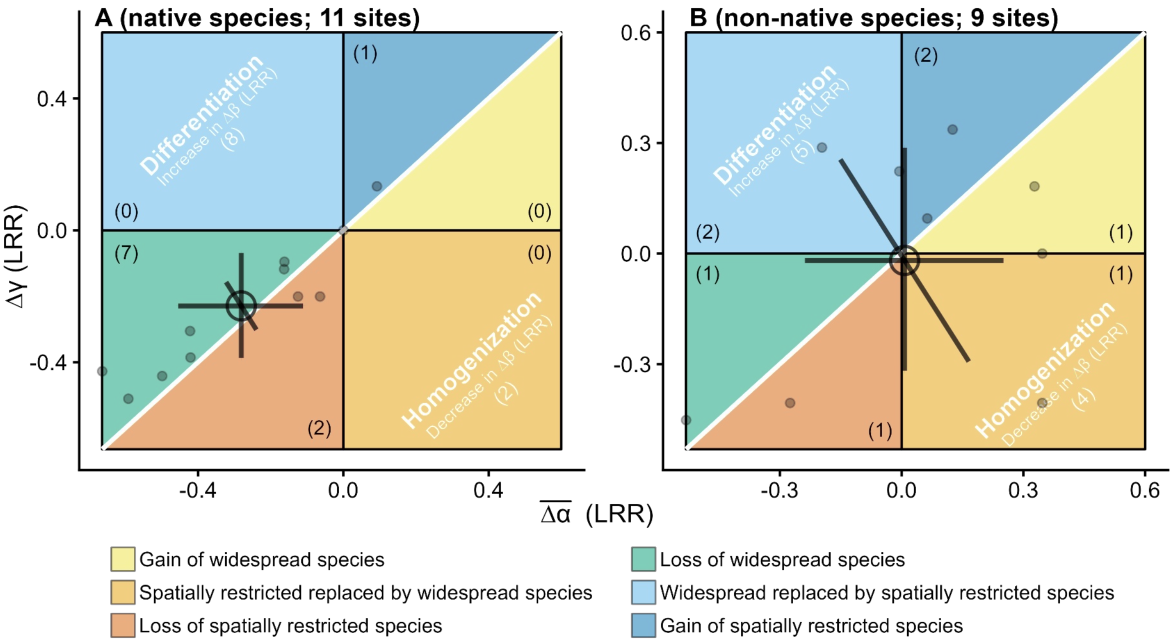

Fig. S9.

Changes in average α, γ, and β diversity ($\overline{\Delta\alpha}$, Δγ, and Δβ) with nutrient addition for native and non-native species groups for 11 sites with five blocks. (A) native and (B) non-native species. LRR: log response ratio. The white 1:1 diagonal line indicates no effects of nutrient addition on β diversity. Numbers in the parentheses are the number of sites. When a site has $\overline{\Delta\alpha}$ = 0, Δγ = 0, or Δβ = 0, it was not counted into any of the six scenarios. The small points represent site-level $\overline{\Delta\alpha}$, Δγ, and Δβ. The large open point and error bars are the estimated mean and 95% credible intervals for $\overline{\Delta\alpha}$, Δγ, and Δβ across all sites. Source data are provided as a Source Data file.

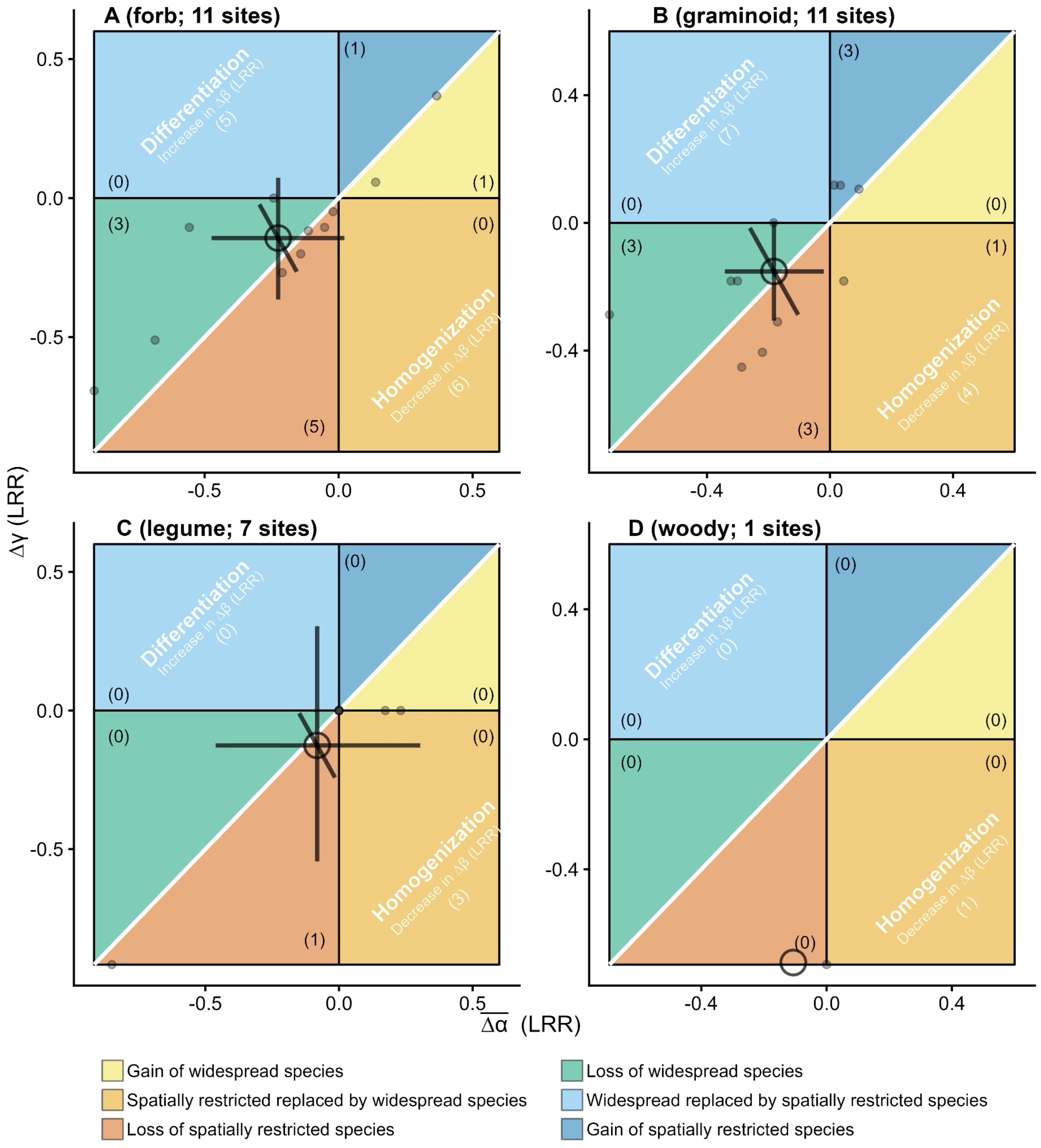

Fig. S10.

Changes in average α, γ, and β diversity ($\overline{\Delta\alpha}$, Δγ, and Δβ) with nutrient addition for different functional species groups for 11 sites with five blocks. (A) forb, (B) graminoid, (C) legume, and (D) woody species. LRR: log response ratio. The white 1:1 diagonal line indicates no effects of nutrient addition on β diversity. Numbers in the parentheses are the number of sites. When a site has $\overline{\Delta\alpha}$ = 0, Δγ = 0, or Δβ = 0, it was not counted into any of the six scenarios. The small points represent site-level $\overline{\Delta\alpha}$, Δγ, and Δβ. The large open point and error bars are the estimated mean and 95% credible intervals for $\overline{\Delta\alpha}$, Δγ, and Δβ across all sites. Source data are provided as a Source Data file.

Table S1.

Site geolocation, grassland types, and experimental years used.

site_code	Continent	Habitat	Latitude	Longitude	First nutrient year	Year used
sereng.tz	Africa	savanna	-2.43	34.86	2009	4
ukul.za	Africa	mesic grassland	-29.67	30.40	2010	4
azi.cn	Asia	alpine grassland	33.67	101.87	2008	4
azitwo.cn	Asia	alpine grassland	33.58	101.53	2019	4
kibber.in	Asia	alpine grassland	32.32	78.01	2012	4
bogong.au	Australia	alpine grassland	-36.87	147.25	2010	4, 14
burrawan.au	Australia	semiarid grassland	-27.74	151.14	2009	4
ethamc.au	Australia	desert grassland	-23.76	138.47	2014	4
ethass.au	Australia	desert grassland	-23.64	138.40	2014	4
kidman.au	Australia	savanna	-16.11	130.95	2015	4
kiny.au	Australia	semiarid grassland	-36.20	143.75	2008	4, 14
mtca.au	Australia	savanna	-31.78	117.61	2009	4
nilla.au	Australia	old field	-36.90	146.01	2017	4
ping.au	Australia	old field	-32.50	116.97	2014	4
yarra.au	Australia	mesic grassland	-33.61	150.74	2015	4
ahth.is	Europe	heathland	65.13	-19.67	2016	4
amlr.is	Europe	desert grassland	65.13	-19.67	2016	4
badlau.de	Europe	old field	51.39	11.88	2016	4
bayr.de	Europe	mesic grassland	49.92	11.58	2017	4
burren.ie	Europe	calcareous grassland	53.07	-8.99	2016	4
comp.pt	Europe	annual grassland	38.83	-8.79	2013	4
frue.ch	Europe	pasture	47.11	8.54	2009	4
hero.uk	Europe	mesic grassland	51.41	-0.64	2008	4, 14
jena.de	Europe	grassland	50.94	11.53	2014	4
kilp.fi	Europe	tundra grassland	69.06	20.87	2014	4
lancaster.uk	Europe	mesic grassland	53.99	-2.63	2009	4
rook.uk	Europe	mesic grassland	51.41	-0.64	2008	4
saana.fi	Europe	montane grassland	69.04	20.84	2015	4
sval.no	Europe	tundra	78.69	16.45	2019	4

		grassland				
thth.is	Europe	heathland	65.90	-17.09	2016	4
tmlr.is	Europe	desert grassland	65.90	-17.09	2016	4
valm.ch	Europe	alpine grassland	46.63	10.37	2009	4
veluwe.nl	Europe	old field	52.03	5.81	2018	4
arch.us	North America	mixedgrass prairie	27.17	-81.22	2016	4
barta.us	North America	mixedgrass prairie	42.24	-99.65	2008	4
bnbt.us	North America	tallgrass prairie	39.60	-95.09	2018	4
bnch.us	North America	montane grassland	44.28	-121.97	2008	4, 14
cbgb.us	North America	tallgrass prairie	41.79	-93.39	2010	4
cdcr.us	North America	tallgrass prairie	45.42	-93.21	2008	4, 14
cdpt.us	North America	shortgrass prairie	41.21	-101.64	2008	4, 14
cowi.ca	North America	old field	48.81	-123.63	2008	4, 14
elliot.us	North America	annual grassland	32.88	-117.05	2009	4, 14
hall.us	North America	tallgrass prairie	36.87	-86.70	2008	4
hart.us	North America	shrub steppe	42.72	-119.50	2008	4
hopl.us	North America	annual grassland	39.01	-123.06	2008	4
kbs.us	North America	old field	42.41	-85.39	2014	4
koffler.ca	North America	pasture	44.02	-79.54	2011	4
konz.us	North America	tallgrass prairie	39.07	-96.58	2008	4, 14
lake.us	North America	tallgrass prairie	43.38	-95.18	2016	4
look.us	North America	montane grassland	44.21	-122.13	2008	4, 14
mcla.us	North America	annual grassland	38.86	-122.41	2008	4
moab.us	North America	NA	38.78	-109.65	2019	4
msla.us	North	grassland	46.66	-114.00	2018	4

	America					
msla_2.us	North America	grassland	46.66	-114.00	2018	4
msla_3.us	North America	grassland	46.66	-114.00	2018	4
msum.us	North America	tallgrass prairie	46.87	-96.45	2017	4
sage.us	North America	montane grassland	39.43	-120.24	2008	4
saline.us	North America	mixedgrass prairie	39.05	-99.10	2008	4
sedg.us	North America	annual grassland	34.70	-120.02	2008	4
sevi.us	North America	desert grassland	34.36	-106.69	2008	4, 14
sgs.us	North America	shortgrass prairie	40.82	-104.77	2008	4, 14
shps.us	North America	shrub steppe	44.26	-112.21	2008	4
sier.us	North America	annual grassland	39.24	-121.28	2008	4
smith.us	North America	mesic grassland	48.21	-122.62	2008	4
spin.us	North America	pasture	38.13	-84.50	2008	4, 14
temple.us	North America	tallgrass prairie	31.04	-97.35	2008	4, 14
trel.us	North America	tallgrass prairie	40.08	-88.83	2009	4
unc.us	North America	old field	36.01	-79.02	2008	4
chilcas.ar	South America	mesic grassland	-36.28	-58.27	2014	4
lagoas.br	South America	cerrado	-20.98	-51.80	2017	4
marc.ar	South America	grassland	-37.72	-57.42	2012	4
potrok.ar	South America	semiarid grassland	-51.92	-70.41	2016	4

Table S2.

Summary of spatial autocorrelation for the effects of nutrient addition on alpha, gamma, and beta diversity (Δα, Δγ, and Δβ) four year after nutrient addition began using Moran's I test. Positive Moran's I suggests similar values cluster together while negative Moran's I suggests dissimilar values are adjacent. The z-score quantifies how extreme the observed Moran's I value is compared with that is expected under the null hypothesis of spatial randomness.

Diversity facets	Moran's I	Standard deviate (z score)	p
Δα	0.1258	1.2920	0.0982
Δγ	0.1274	1.3251	0.0926
Δβ	-0.0312	-0.1687	0.5670

Table S3.

Summary of model fit for the effects of nutrient addition on alpha, gamma, and beta diversity (Δα, Δγ, and Δβ) four years after treatment began. We calculated α and γ diversity using species richness, effective number of species (ENS) based on Shannon diversity, and Simpson diversity. We fitted the random intercept models using the brm function. For Δα, Δγ, and Δβ, we included random intercepts among sites, model was coded as: diversity (log scale) ~ 1 + (1 |sites). Bulk-ESS is the effective sample size, it is useful as a diagnostic for the sampling efficiency. Tail_ESS is the minimum of the effective sample sizes for 5% and 95% quantiles. Low Rhats indicates model convergence were achieved.

Diversity metrics	Diversity facets	Estimate	l-95% CI	u-95% CI	Rhat	Bulk_ESS	Tail_ESS	Number of sites
Species richness	Δα	-0.1916	-0.2549	-0.1297	1.0013	16067.290	9466.034	72
Species richness	Δβ	0.0280	-0.0208	0.0763	1.0027	2133.078	1718.600	72
Species richness	Δγ	-0.1623	-0.2365	-0.0848	1.0040	1583.065	1456.179	72
Simpson diversity	Δα	-0.1833	-0.2611	-0.1062	1.0006	11229.847	9428.506	72
Simpson diversity	Δβ	0.0083	-0.0599	0.0767	1.0013	3977.223	5845.292	72
Simpson diversity	Δγ	-0.1768	-0.2896	-0.0616	1.0019	2636.618	5521.383	72
Shannon diversity	Δα	-0.2027	-0.2802	-0.1269	1.0001	7935.751	8781.933	72
Shannon diversity	Δβ	0.0102	-0.0423	0.0632	1.0028	2269.188	3430.052	72
Shannon diversity	Δγ	-0.1933	-0.2953	-0.0919	1.0022	2596.788	3157.553	72

Table S4.

Estimated mean and 95% credible intervals for change in the average alpha, gamma, and beta diversity ($\overline{\Delta\alpha}$, Δγ, and Δβ) at individual sites four years under nutrient addition began. Diversity was measured as species richness. Scenarios differed from that described in the main text because here we considered the 95% credible intervals estimated from models. Sites where $\overline{\Delta\alpha}$, Δγ, or Δβ overlaps with 0 were not assigned to any of the six scenarios in the framework and were categorized as 'Other scenarios'.

site_code	Δα [l-95% CI, u-95% CI]	Δγ [l-95% CI, u-95% CI]	Δβ [l-95% CI, u-95% CI]	Change in widespread or spatially restricted species
ahth.is	-0.1513 [-0.3345, 0.0898]	-0.0018 [-0.2977, 0.2935]	0.0524 [-0.1133, 0.2255]	Other situations
amlr.is	-0.1293 [-0.3044, 0.1305]	0.0195 [-0.2862, 0.3364]	-0.004 [-0.1752, 0.1597]	Other situations
arch.us	-0.1937 [-0.394, 0.0081]	-0.1352 [-0.3858, 0.1129]	0.0588 [-0.1064, 0.2306]	Other situations
azi.cn	-0.1797 [-0.3702, 0.0275]	-0.1103 [-0.3606, 0.145]	0.038 [-0.1251, 0.2042]	Other situations
azitwo.cn	-0.2559 [-0.5218, -0.0785]	-0.3898 [-0.7254, -0.0557]	0.0193 [-0.1472, 0.1824]	Loss of spatially restricted species
badlau.de	-0.1815 [-0.3796, 0.0313]	-0.1661 [-0.413, 0.0768]	-0.0104 [-0.185, 0.154]	Other situations
barta.us	-0.2067 [-0.4218, -0.0162]	-0.2124 [-0.4626, 0.0391]	0.0328 [-0.1301, 0.1974]	Other situations
bayr.de	-0.1721 [-0.3595, 0.0459]	-0.1245 [-0.3836, 0.1291]	0.0041 [-0.1662, 0.167]	Other situations
bnbt.us	-0.171 [-0.3579, 0.0509]	-0.0852 [-0.3386, 0.183]	0.0357 [-0.1256, 0.2019]	Other situations
bnch.us	-0.1835 [-0.3783, 0.0256]	-0.1495 [-0.4095, 0.1021]	0.0171 [-0.1529, 0.1817]	Other situations
bogong.au	-0.1724 [-0.3579, 0.0397]	-0.0607 [-0.3263, 0.2094]	0.063 [-0.104, 0.2376]	Other situations
burrawan.au	-0.2235 [-0.4485, -0.0414]	-0.0855 [-0.3425, 0.1832]	0.1973 [-0.0297, 0.4582]	Other situations
burren.ie	-0.2622 [-0.5309, -0.0873]	-0.3326 [-0.6436, -0.0315]	0.0903 [-0.0789, 0.2777]	Loss of spatially restricted species
cbgb.us	-0.178 [-0.3726, 0.0382]	-0.1813 [-0.4297, 0.0712]	-0.0292 [-0.2092, 0.1388]	Other situations
cdcr.us	-0.2813 [-0.5773, -0.101]	-0.2129 [-0.467, 0.0364]	0.2582 [-0.0123, 0.5784]	Other situations
cdpt.us	-0.2165 [-0.4429, -0.0294]	-0.2818 [-0.5598, -0.0101]	0.0014 [-0.1685, 0.1683]	Loss of spatially restricted species
chilcas.ar	-0.183 [-0.3801,	-0.6158 [-1.1399,	-0.408 [-0.9449,	Other situations

site_code	Δα [l-95% CI, u-95% CI]	Δγ [l-95% CI, u-95% CI]	Δβ [l-95% CI, u-95% CI]	Change in widespread or spatially restricted species
	0.0232]	-0.1047]	0.0524]	
comp.pt	-0.2074 [-0.4253, -0.0128]	-0.1502 [-0.396, 0.0948]	0.0906 [-0.0763, 0.2792]	Other situations
cowi.ca	-0.228 [-0.4582, -0.05]	-0.4199 [-0.7836, -0.0737]	-0.0956 [-0.3162, 0.0988]	Loss of spatially restricted species
elliot.us	-0.2713 [-0.5577, -0.0976]	-0.299 [-0.5896, -0.02]	0.1546 [-0.0396, 0.3784]	Loss of spatially restricted species
ethamc.au	-0.1729 [-0.357, 0.0382]	-0.0851 [-0.3493, 0.1888]	0.0415 [-0.1184, 0.2063]	Other situations
ethass.au	-0.099 [-0.2788, 0.2066]	0.3106 [-0.2176, 0.8525]	0.1722 [-0.032, 0.4095]	Other situations
frue.ch	-0.2021 [-0.4151, -0.008]	-0.1624 [-0.4027, 0.0876]	0.057 [-0.106, 0.2292]	Other situations
hall.us	-0.1647 [-0.3506, 0.0631]	-0.0837 [-0.3424, 0.1758]	0.0172 [-0.1511, 0.1812]	Other situations
hart.us	-0.1532 [-0.3296, 0.0811]	-0.0257 [-0.3082, 0.255]	0.0329 [-0.1316, 0.2015]	Other situations
hero.uk	-0.1799 [-0.3744, 0.0321]	-0.0154 [-0.3022, 0.2698]	0.1258 [-0.0578, 0.3283]	Other situations
hopl.us	-0.254 [-0.515, -0.0751]	-0.3385 [-0.6433, -0.0408]	0.0625 [-0.1024, 0.2365]	Loss of spatially restricted species
jena.de	-0.165 [-0.3477, 0.0586]	-0.085 [-0.3421, 0.1809]	0.0112 [-0.1609, 0.1715]	Other situations
kbs.us	-0.2452 [-0.4933, -0.0684]	-0.3971 [-0.7384, -0.0627]	-0.0141 [-0.1881, 0.1459]	Loss of spatially restricted species
kibber.in	-0.1677 [-0.3563, 0.0563]	-0.1305 [-0.3816, 0.1251]	-0.0166 [-0.1962, 0.1491]	Other situations
kidman.au	-0.148 [-0.3254, 0.0941]	0.0958 [-0.2537, 0.4568]	0.1309 [-0.0511, 0.3364]	Other situations
kilp.fi	-0.1849 [-0.3804, 0.0205]	-0.1291 [-0.3762, 0.1214]	0.0446 [-0.1193, 0.214]	Other situations
kiny.au	-0.1923 [-0.3947, 0.0125]	-0.1329 [-0.3791, 0.1185]	0.0582 [-0.1084, 0.2351]	Other situations
koffler.ca	-0.2522 [-0.5119, -0.0763]	-0.2996 [-0.5921, -0.0197]	0.0935 [-0.0748, 0.2781]	Loss of spatially restricted species
konz.us	-0.1688 [-0.3615, 0.0577]	-0.0837 [-0.3409, 0.1872]	0.0258 [-0.1356, 0.1831]	Other situations
lagoas.br	-0.2405 [-0.4854, -0.0665]	-0.2798 [-0.5651, -0.0048]	0.073 [-0.0908, 0.2506]	Loss of spatially restricted species
lake.us	-0.2153 [-0.4433, -0.0263]	-0.1695 [-0.4234, 0.0776]	0.093 [-0.0757, 0.2747]	Other situations

site_code	Δα [l-95% CI, u-95% CI]	Δγ [l-95% CI, u-95% CI]	Δβ [l-95% CI, u-95% CI]	Change in widespread or spatially restricted species
lancaster.uk	-0.1846 [-0.3794, 0.0152]	-0.1636 [-0.4148, 0.0872]	0.0053 [-0.1683, 0.1695]	Other situations
look.us	-0.1815 [-0.38, 0.0307]	-0.1719 [-0.4231, 0.0826]	-0.0162 [-0.1977, 0.1507]	Other situations
marc.ar	-0.1834 [-0.3785, 0.0248]	-0.1038 [-0.3609, 0.1523]	0.0562 [-0.1093, 0.228]	Other situations
mcla.us	-0.1632 [-0.3509, 0.0622]	-0.0414 [-0.3211, 0.2397]	0.054 [-0.1063, 0.2249]	Other situations
moab.us	-0.1804 [-0.3769, 0.0311]	-0.0849 [-0.3485, 0.1851]	0.0638 [-0.0966, 0.2384]	Other situations
msla.us	-0.1838 [-0.3788, 0.0248]	-0.1041 [-0.3631, 0.1584]	0.0556 [-0.107, 0.2263]	Other situations
msla_2.us	-0.1501 [-0.3347, 0.0899]	-0.0262 [-0.3136, 0.2586]	0.0176 [-0.1505, 0.1787]	Other situations
msla_3.us	-0.1707 [-0.3606, 0.0551]	-0.1199 [-0.3677, 0.136]	-0.0003 [-0.1687, 0.1624]	Other situations
msum.us	-0.2032 [-0.4143, -0.0121]	-0.1344 [-0.3806, 0.1163]	0.09 [-0.0808, 0.2761]	Other situations
mtca.au	-0.2406 [-0.4864, -0.0683]	-0.4201 [-0.7744, -0.0744]	-0.0554 [-0.2506, 0.1167]	Loss of spatially restricted species
nilla.au	-0.1286 [-0.3056, 0.1346]	0.0428 [-0.275, 0.3674]	0.023 [-0.1441, 0.188]	Other situations
ping.au	-0.2343 [-0.4763, -0.0482]	-0.2589 [-0.5213, -0.0009]	0.0724 [-0.091, 0.2491]	Loss of spatially restricted species
potrok.ar	-0.1573 [-0.3446, 0.0769]	-0.1116 [-0.3635, 0.1501]	-0.031 [-0.2136, 0.1391]	Other situations
rook.uk	-0.2072 [-0.4198, -0.0185]	-0.0478 [-0.3141, 0.2278]	0.1817 [-0.0288, 0.4256]	Other situations
saana.fi	-0.2274 [-0.4557, -0.0494]	-0.337 [-0.6469, -0.0354]	-0.0192 [-0.1964, 0.1462]	Loss of spatially restricted species
sage.us	-0.1643 [-0.3448, 0.0652]	-0.0636 [-0.3271, 0.2027]	0.0358 [-0.1274, 0.1971]	Other situations
saline.us	-0.203 [-0.4175, -0.009]	-0.1918 [-0.4408, 0.0577]	0.0349 [-0.1288, 0.1997]	Other situations
sedg.us	-0.26 [-0.5265, -0.0829]	-0.6189 [-1.1423, -0.1064]	-0.1708 [-0.4567, 0.0734]	Loss of spatially restricted species
sereng.tz	-0.1746 [-0.3664, 0.039]	-0.1116 [-0.3689, 0.1445]	0.0252 [-0.1423, 0.191]	Other situations
sevi.us	-0.1723 [-0.3658, 0.0487]	-0.172 [-0.4239, 0.0771]	-0.0342 [-0.2206, 0.1343]	Other situations
sgs.us	-0.1392 [-0.317,	-0.1853 [-0.4385,	-0.1543 [-0.427,	Other situations

site_code	Δα [l-95% CI, u-95% CI]	Δγ [l-95% CI, u-95% CI]	Δβ [l-95% CI, u-95% CI]	Change in widespread or spatially restricted species
	0.1092]	0.0647]	0.0818]	
shps.us	-0.2123 [-0.4319, -0.0174]	-0.253 [-0.5209, 0.0051]	0.0137 [-0.1593, 0.1798]	Other situations
sier.us	-0.1125 [-0.2864, 0.1655]	0.1126 [-0.2536, 0.4853]	0.0333 [-0.1335, 0.198]	Other situations
smith.us	-0.1754 [-0.3656, 0.0435]	-0.1661 [-0.4131, 0.0835]	-0.0221 [-0.1992, 0.1432]	Other situations
spin.us	-0.217 [-0.4405, -0.0307]	-0.1363 [-0.39, 0.1178]	0.131 [-0.0485, 0.3353]	Other situations
sval.no	-0.1567 [-0.3434, 0.0771]	-0.0415 [-0.3041, 0.2327]	0.033 [-0.1319, 0.1987]	Other situations
temple.us	-0.2157 [-0.4384, -0.0235]	-0.2568 [-0.5186, 0.0019]	0.0159 [-0.1504, 0.1824]	Other situations
thth.is	-0.194 [-0.402, 0.0034]	-0.1157 [-0.3701, 0.1447]	0.0824 [-0.0821, 0.259]	Other situations
tmlr.is	-0.1677 [-0.36, 0.0535]	-0.1196 [-0.3681, 0.136]	-0.0005 [-0.1752, 0.1639]	Other situations
trel.us	-0.1992 [-0.4047, -0.0041]	-0.1906 [-0.4477, 0.0595]	0.0225 [-0.1443, 0.189]	Other situations
ukul.za	-0.1777 [-0.3676, 0.038]	-0.1006 [-0.3562, 0.1626]	0.0406 [-0.122, 0.2095]	Other situations
unc.us	-0.253 [-0.5182, -0.0774]	-0.4709 [-0.8686, -0.082]	-0.0596 [-0.2601, 0.1169]	Loss of spatially restricted species
valm.ch	-0.2081 [-0.423, -0.014]	-0.2233 [-0.4889, 0.0354]	0.0258 [-0.1414, 0.1936]	Other situations
veluwe.nl	-0.1485 [-0.3297, 0.0946]	-0.0172 [-0.2972, 0.2769]	0.0233 [-0.1452, 0.1843]	Other situations
yarra.au	-0.2078 [-0.4271, -0.0132]	-0.3555 [-0.6704, -0.0414]	-0.0961 [-0.3189, 0.0981]	Loss of spatially restricted species

Table S5.
Summary of model fit for the effects of nutrient addition on alpha, gamma, and beta diversity (Δα, Δγ, and Δβ) for native and non-native species four years after treatment began. We calculated α and γ diversity using species richness. We fitted the random intercept models using the brm function. For Δα, Δγ, and Δβ, we included random intercepts among sites, model was coded as: diversity (log scale) ~ 1 + (1 |sites). Bulk-ESS is the effective sample size, it is useful as a diagnostic for the sampling efficiency. Tail_ESS is the minimum of the effective sample sizes for 5% and 95% quantiles. Low Rhats indicates model convergence were achieved.

Species groups	Diversity facets	Estimate	l-95% CI	u-95% CI	Rhat	Bulk_ESS	Tail_ESS	Number of sites
Native	Δα	-0.2118	-0.2861	-0.1374	1.0009	12510.4909	8656.293	69
Native	Δβ	0.0005	-0.0548	0.0552	1.0172	342.3685	806.045	69
Native	Δγ	-0.2113	-0.3007	-0.1234	1.0033	2001.1538	3930.769	69
Non-native	Δα	-0.1155	-0.2278	-0.0038	1.0007	9704.4354	8918.270	42
Non-native	Δβ	0.0454	-0.0466	0.1380	1.0020	2009.6608	1466.581	42
Non-native	Δγ	-0.0499	-0.1972	0.1015	1.0046	1921.3336	2824.314	42

Table S6.
Summary of model fit for the effects of nutrient addition on alpha, gamma, and beta diversity (Δα, Δγ, and Δβ) for different life forms four years after treatment began. We calculated α and γ diversity using species richness. We fitted the random intercept models using the brm function. For Δα, Δγ, and Δβ, we included random intercepts among sites, model was coded as: diversity (log scale) ~ 1 + (1 |sites). Bulk-ESS is the effective sample size, it is useful as a diagnostic for the sampling efficiency. Tail_ESS is the minimum of the effective sample sizes for 5% and 95% quantiles. Low Rhats indicates model convergence were achieved.

Species groups	Diversity facets	Estimate	l-95% CI	u-95% CI	Rhat	Bulk_ESS	Tail_ESS	Number of sites
FORB	Δα	-0.1941	-0.2908	-0.0939	1.0009	10299.512 1	8854.6421	68
FORB	Δβ	0.0854	-0.0155	0.1874	1.0054	1579.1276	3469.0268	68
FORB	Δγ	-0.1139	-0.2223	-0.0125	1.0254	191.9344	4084.4711	68
GRAMINOID	Δα	-0.1320	-0.1998	-0.0628	1.0005	10726.078 3	8985.3000	72
GRAMINOID	Δβ	0.0061	-0.0393	0.0546	1.0038	1644.1281	1257.2676	72
GRAMINOID	Δγ	-0.1276	-0.2091	-0.0476	1.0060	2455.7868	4956.4376	72
LEGUME	Δα	-0.1894	-0.3169	-0.0600	1.0010	12678.770 8	8585.2324	34
LEGUME	Δβ	0.0049	-0.1681	0.1759	1.0009	3779.7706	4843.1788	34
LEGUME	Δγ	-0.1887	-0.3895	-0.0029	1.0095	703.4521	708.2061	34
WOODY	Δα	-0.1828	-0.3425	-0.0197	1.0005	8915.9640	7764.9732	23
WOODY	Δβ	-0.1426	-0.2950	0.0027	1.0016	2747.1866	3021.2355	23
WOODY	Δγ	-0.2887	-0.5220	-0.0563	1.0027	1919.7104	2497.4429	23

Table S7.

Principal investigators contributing data but are not authors; Names match those in Table S1. Their effort in providing data is critical to this manuscript.

site_code	PI name	Institution
ahth.is	Isabel Barrio	University of Iceland
amlr.is	Isabel Barrio	University of Iceland
arch.us	Elizabeth Boughton	MacArthur Agro-ecology Research Center
azi.cn	Chengjin Chu	Lanzhou University
azi.cn	Guozhen Du	Lanzhou University
azi.cn	Qi Li	Northwest Institute of Plateau Biology
azi.cn	Wei Li	Iowa State University
azi.cn	Gang Wen	Lanzhou University
azitwo.cn	Guozhen Du	Lanzhou University
azitwo.cn	Zhengwei Ren	Lanzhou University
badlau.de	Julia Siebert	German Centre for Integrative Biodiversity Research (iDiv)
barta.us	David Wedin	University of Nebraska, Lincoln
bayr.de	Marie Spohn	NULL
bnbt.us	Brent Mortensen	Benedictine College
bnbt.us	Janet Paper	Benedictine College
bogong.au	Joslin Moore	Victoria State Government
burrawan.au	Jennifer Firn	Queensland University of Technology
burren.ie	Yvonne Buckley	Trinity College Dublin, the University of Dublin
cbgb.us	Lori Biederman	Iowa State University
cbgb.us	Kirsten Hofmockel	Iowa State University
cbgb.us	Lauren Sullivan	Iowa State University
cdcr.us	Adam Kay	University of St. Thomas
chilcas.ar	Enrique Chaneton	Universidad de Buenos Aires
chilcas.ar	Laura Yahdjian	Universidad de Buenos Aires
comp.pt	Maria Caldeira	Technical University of Lisbon
elliot.us	Elsa Cleland	University of California, San Diego
frue.ch	Sabine Güsewell	ETH Zurich
frue.ch	Andy Hector	University of Zurich
hall.us	Rebecca McCulley	University of Kentucky
hall.us	Jim Nelson	University of Kentucky
hart.us	Nicole DeCrappeo	USGS
hart.us	David Pyke	USGS
hero.uk	Mick Crawley	Imperial College at Silwood Park
jena.de	Anne Ebeling	Friedrich-Schiller-Universität Jena
kbs.us	Lars Brudvig	Michigan State University
kibber.in	Mahesh Sankaran	National Centre for Biological Sciences
kidman.au	Anna Richards	CSIRO
koffler.ca	Arthur Weiss	University of Toronto Scarborough
konz.us	Kimberly Komatsu	University of North Carolina - Greensboro

site_code	PI name	Institution
konz.us	Melinda Smith	Colorado State University
lagoas.br	Lucíola Lannes	Universidade Estadual Paulista - UNESP
lake.us	Lori Biederman	Iowa State University
marc.ar	Juan Alberti	Universidad Nacional de Mar del Plata
moab.us	Brooke Osborne	US Geological Survey
moab.us	Sasha Reed	US Geological Survey
msla.us	Mary Ellyn DuPre	MPG Ranch
msla.us	Kelly Laflamme	MPG Ranch
msla.us	Yiva Lekberg	MPG Ranch
msla_2.us	Mary Ellyn DuPre	MPG Ranch
msla_2.us	Yiva Lekberg	MPG Ranch
msla_3.us	Mary Ellyn DuPre	MPG Ranch
msla_3.us	Yiva Lekberg	MPG Ranch
msum.us	Alison Wallace	Minnesota State University Moorhead
mtca.au	Suzanne Prober	CSIRO
ping.au	Jodi Price	The University of Western Australia
ping.au	Rachel Standish	The University of Western Australia
potrok.ar	Hector Bahamonde	UNPA - CONICET
potrok.ar	Pablo Peri	UNPA - CONICET
rook.uk	Mick Crawley	Imperial College at Silwood Park
sage.us	Louie Yang	University of California, Davis
saline.us	Kimberly Komatsu	University of North Carolina - Greensboro
saline.us	Melinda Smith	Colorado State University
sereng.tz	T. Anderson	Wake Forest University
sevi.us	Scott Collins	University of New Mexico
sevi.us	Laura Ladwig	University of New Mexico
sgs.us	Dana Blumenthal	USDA-ARS
sgs.us	Cynthia Brown	Colorado State University
sgs.us	Julia Klein	Colorado State University
sgs.us	Alan Knapp	Colorado State University
smith.us	Janneke Hille Ris Lambers	University of Washington
spin.us	Rebecca McCulley	University of Kentucky
spin.us	Jim Nelson	University of Kentucky
temple.us	Philip Fay	USDA ARS
temple.us	Jason Martina	Texas State University
thth.is	Isabel Barrio	University of Iceland
tmlr.is	Isabel Barrio	University of Iceland
trel.us	Andrew Leakey	University of Illinois at Urbana-Champaign
ukul.za	Kevin Kirkman	University of KwaZulu-Natal
unc.us	Charles Mitchell	University of North Carolina
unc.us	Justin Wright	Duke University
yarra.au	Raul Ochoa Hueso	University of Western Sydney

Table S8.
Contributions of each author to the manuscript. Author contributions to individual tasks are marked with x.

Full name	Sites used in analysis	Developed and framed research questions	Analyzed data	Contributed to data analyses	Wrote the paper	Contributed to paper writing	Site coordinator	Nutrient Network coordinator	Site level acknowledgments
Qingqing Chen		x	x		x				
Shane Blowes		x		x		x			
Emma Ladouceur		x		x		x			
W. Stanley Harpole	hopl.us, mcla.us, sier.us, cbgb.us					x	x	x	
Jonathan Chase		x				x			
Jonathan D. Bakker	smith.us			x		x	x		
Nico Eisenhauer	badlau.de					x	x		NE acknowledges funding by the German Research Foundation (DFG, FZT 118, 202548816; Ei 862/29-1).
Eric W. Seabloom	bnch.us, look.us, hopl.us, mcla.us, sier.us, cdcr.us					x	x	x	
Pablo L. Peri	potrok.ar					x	x		
Pedro M. Tognetti	chilcas.ar					x	x		
Risto Virtanen	kilp.fi,saana.fi					x	x		
Sally A. Power	Yarra.au					x	x		

Full name	Sites used in analysis	Developed and framed research questions	Analyzed data	Contributed to data analyses	Wrote the paper	Contributed to paper writing	Site coordinator	Nutrient Network coordinator	Site level acknowledgments
George R. Wheeler	cdpt.us					x	x		
Michelle Tedder	ukul.za, gilbza					x	x		
Anita C. Risch	valm.ch					x	x		
Sumanta Bagchi	kibber.in								
Jane A. Catford	nilla.au					x	x		
Carly J. Stevens	lancaster.uk								
Christiane Roscher	jena.de					x	x		
Yujie Niu	bayr.de					x			
Sylvia Haider	badlau.de					x	x		
Maria C. Caldeira	comp.pt					x	x		to Companhia das Lezirias for granting access to the study site and Portuguese Science Foundation (FCT) for Forest Research Centre funding (UIDB/00239/2020).
Peter B. Adler	shps.us					x	x		
Jason P. Martina	temple.us					x	x		
Miguel N. Bugalho	comp.pt					x	x		
Johannes M H Knops	cdpt.us					x	x		
Elizabeth T. Borer	bnch.us, look.us, hopl.us, mcla.us, sier.us, cdcr.us					x	x	x	

Full name	Sites used in analysis	Developed and framed research questions	Analyzed data	Contributed to data analyses	Wrote the paper	Contributed to paper writing	Site coordinator	Nutrient Network coordinator	Site level acknowledgments
Christopher R. Dickman	ethamc.au, ethass.au					x	x		
Anke Jentsch	bayr.de					x	x		AJ acknowledges funding by the Federal Ministry of Education and Research (BMBF) grant 031B0516C and by the Upper Franconian Trust Oberfrankenstiftung grant FP00237
Yann Hautier	frue.ch					x	x		
Andrew MacDougall	Cowi.ca					x	x		
John W. Morgan	kiny.au, bogong.au					x	x		
Glenda M. Wardle	ethamc.au, ethass.au					x	x		GMW acknowledges Bush Heritage Australia and the Wangkamadla people for access to the sites and the ARC, and TERN funded by NCRIS for funding
Pedro Daleo	marc.ar					x	x		
Catalina Estrada	hero.uk, rook.uk					x	x		Imperial College London, Department of Life Sciences
Ian Donohue	burren.ie					x	x		
G.F. (Ciska) Veen	veluwe.nl					x	x		
Daniel S Gruner	sage.us					x	x		
Harry Olde Venterink	lagoas.br					x	x		
Erika Hersch-Green						x			
Anu Eskelinen	kilp.fi					x	x		

**Full name**	**Sites used in analysis**	**Developed and framed research questions**	**Analyzed data**	**Contributed to data analyses**	**Wrote the paper**	**Contributed to paper writing**	**Site coordinator**	**Nutrient Network coordinator**	**Site level acknowledgments**
Nicole Hagenah	ukul.za					x	x		
Carla D'Antonio	sedg.us					x	x		
Marc W. Cadotte	Koffler					x	x		
Brooke B. Osborne	moab.us					x	x		
Lauri Laanisto	sval.no					x	x		Funded by Estonian Academy of Sciences (research professorship for Arctic studies)
Petr Macek	sval.no					x	x		Funded by Estonian Academy of Sciences (research professorship for Arctic studies)
