# Supplementary material for: Local nutrient addition drives plant diversity losses but not biotic homogenization in global grasslands
Source: Nat Commun. 2025 May 27;16:4903. doi: 10.1038/s41467-025-59166-7 (PMC12116774; doi:10.1038/s41467-025-59166-7)
Supplement: Supplementary file 3 — Reporting Summary [file 41467_2025_59166_MOESM3_ESM.pdf]

nature portfolio

Corresponding author(s): Qingqing Chen

Last updated by author(s): Feb 18, 2025

Reporting Summary

Nature Portfolio wishes to improve the reproducibility of the work that we publish. This form provides structure for consistency and transparency in reporting. For further information on Nature Portfolio policies, see our Editorial Policies and the Editorial Policy Checklist.

Statistics

For all statistical analyses, confirm that the following items are present in the figure legend, table legend, main text, or Methods section.

n/a	Confirmed	
☐	☒	The exact sample size (*n*) for each experimental group/condition, given as a discrete number and unit of measurement
☐	☒	A statement on whether measurements were taken from distinct samples or whether the same sample was measured repeatedly
☐	☒	The statistical test(s) used AND whether they are one- or two-sided<br>*Only common tests should be described solely by name; describe more complex techniques in the Methods section.*
☐	☒	A description of all covariates tested
☐	☒	A description of any assumptions or corrections, such as tests of normality and adjustment for multiple comparisons
☐	☒	A full description of the statistical parameters including central tendency (e.g. means) or other basic estimates (e.g. regression coefficient) AND variation (e.g. standard deviation) or associated estimates of uncertainty (e.g. confidence intervals)
☒	☐	For null hypothesis testing, the test statistic (e.g. *F*, *t*, *r*) with confidence intervals, effect sizes, degrees of freedom and *P* value noted<br>*Give P values as exact values whenever suitable.*
☐	☒	For Bayesian analysis, information on the choice of priors and Markov chain Monte Carlo settings
☐	☒	For hierarchical and complex designs, identification of the appropriate level for tests and full reporting of outcomes
☐	☒	Estimates of effect sizes (e.g. Cohen's *d*, Pearson's *r*), indicating how they were calculated

Our web collection on statistics for biologists contains articles on many of the points above.

Software and code

Policy information about availability of computer code

Data collection

We used experimental data from 72 grassland sites that are part of the Nutrient Network (NutNet) Global Research Cooperative. See http://www.nutnet.org for more details for this network. Data were retrieved from the NutNet database in November 2023. We analyzed data from 72 sites where 1) nutrients were applied for at least four years; and 2) each site had at least three blocks. These sites are distributed across six continents and include a wide range of grassland types. Sites cover 6 continents and 12 countries, and a wide range of grassland types. For the analyses here, we use two treatments: Ambient (Control) and fertilization by nitrogen, phosphorus, and potassium together (i.e., NPK). Treatments were randomly assigned to 5 m × 5 m plots and were replicated in three or more blocks. Species cover was recorded in one 1 m × 1 m permanent subplot using a standardized protocol. We used data collected at four years and a subset at 14 years after nutrient additions began. The species cover and species richness data, site abiotic and biotic environmental data used and generated in this study have been deposited in the Figshare database and are publicly available (https://doi.org/10.6084/m9.figshare.26412295.v3). The NutNet data are publicly available on the Environmental Data Initiative (EDI) (https://portal.edirepository.org/nis/advancedSearch.jsp).

Data analysis

At each site, α diversity is determined as the number of species in each permanent subplot (i.e., species richness), and γ diversity as the total number of species occurring in three permanent subplots (for each treatment separately). We exclude additional blocks from sites that have more than three because γ and β diversity depend on the number of local communities used. We calculate Δα as the richness difference in local communities (subplots) and Δγ as the difference in the sum of the subplots under nutrient addition relative to that of control on the log scale. We then calculate Δβ as Δγ minus the average of Δα over three blocks. We fitted multilevel (also referred as mixed effects or hierarchical) models for Δα, Δγ, and Δβ (as the response variable; all on the log scale) separately. We included random intercept for each site, model was coded as: richness change ~ 1 + (1 |sites) to estimate site-level variation. The R codes used to produce results in this study have been deposited in the GitHub (https://github.com/chqq365/plant-diversity-and-biotic-homogenization.git) and archived through Zenodo (https://doi.org/10.5281/zenodo.14902812).

For manuscripts utilizing custom algorithms or software that are central to the research but not yet described in published literature, software must be made available to editors and reviewers. We strongly encourage code deposition in a community repository (e.g. GitHub). See the Nature Portfolio guidelines for submitting code & software for further information.

Data

Policy information about availability of data

All manuscripts must include a data availability statement. This statement should provide the following information, where applicable:

- Accession codes, unique identifiers, or web links for publicly available datasets
- A description of any restrictions on data availability
- For clinical datasets or third party data, please ensure that the statement adheres to our policy

The species cover and species richness data, site abiotic and biotic environmental data used and generated in this study have been deposited in the Figshare database and are publicly available (https://doi.org/10.6084/m9.figshare.26412295.v3). The NutNet data are publicly available on the Environmental Data Initiative (EDI) (https://portal.edirepository.org/nis/advancedSearch.jsp).

Research involving human participants, their data, or biological material

Policy information about studies with human participants or human data. See also policy information about sex, gender (identity/presentation), and sexual orientation and race, ethnicity and racism.

Reporting on sex and gender

Use the terms sex (biological attribute) and gender (shaped by social and cultural circumstances) carefully in order to avoid confusing both terms. Indicate if findings apply to only one sex or gender; describe whether sex and gender were considered in study design; whether sex and/or gender was determined based on self-reporting or assigned and methods used.
Provide in the source data disaggregated sex and gender data, where this information has been collected, and if consent has been obtained for sharing of individual-level data; provide overall numbers in this Reporting Summary. Please state if this information has not been collected.
Report sex- and gender-based analyses where performed, justify reasons for lack of sex- and gender-based analysis.

Reporting on race, ethnicity, or other socially relevant groupings

Please specify the socially constructed or socially relevant categorization variable(s) used in your manuscript and explain why they were used. Please note that such variables should not be used as proxies for other socially constructed/relevant variables (for example, race or ethnicity should not be used as a proxy for socioeconomic status).
Provide clear definitions of the relevant terms used, how they were provided (by the participants/respondents, the researchers, or third parties), and the method(s) used to classify people into the different categories (e.g. self-report, census or administrative data, social media data, etc.)
Please provide details about how you controlled for confounding variables in your analyses.

Population characteristics

Describe the covariate-relevant population characteristics of the human research participants (e.g. age, genotypic information, past and current diagnosis and treatment categories). If you filled out the behavioural & social sciences study design questions and have nothing to add here, write "See above."

Recruitment

Describe how participants were recruited. Outline any potential self-selection bias or other biases that may be present and how these are likely to impact results.

Ethics oversight

Identify the organization(s) that approved the study protocol.

Note that full information on the approval of the study protocol must also be provided in the manuscript.

Field-specific reporting

Please select the one below that is the best fit for your research. If you are not sure, read the appropriate sections before making your selection.

☐ Life sciences ☐ Behavioural & social sciences ☒ Ecological, evolutionary & environmental sciences

For a reference copy of the document with all sections, see nature.com/documents/nr-reporting-summary-flat.pdf

Ecological, evolutionary & environmental sciences study design

All studies must disclose on these points even when the disclosure is negative.

Study description	The 72 experimental sites used in this study are part of the Nutrient Network (NutNet). The experimental design includes a factorial manipulation of nutrients (N, P, and K) plus two fences to exclude herbivores. For the analyses here, we used plots under two treatments: Ambient (Control) and fertilization by nitrogen, phosphate, and potassium together (i.e., NPK). A micronutrient mix consists of Fe (15%), S (14%), Mg (1.5%), Mn (2.5%), Cu (1%), Zn (1%), B (0.2%), and Mo (0.05%) was added once only at the start of the experiment (i.e., year 1) for the nutrient addition plots, but not in subsequent years to avoid toxicity. Nitrogen, phosphate, potassium were added annually before the growing season of each treatment year at most sites. Nitrogen was added as 10gm−2yr −1 time-release urea [(NH2)2CO], phosphate was added as 10gm−2yr−1 triple-super phosphate [Ca(H2PO4)2], while potassium was added as 10gm−2yr−1 sulfate [K2SO4].
Research sample	Site PIs recorded percent cover for individual plant species, species richness, and sort them into native and non-native species as well as into life forms (forbs, woody, graminoid and legume species) from 72 grassland sites across the globe (https://nutnet.org/).
Sampling strategy	A 1 ×1 m subplot within each plot was permanently marked for annual measurement of plant community composition. Sampling was done in 1 m2 subplots and followed a standardized protocol at all sites. The number of samples was chosen to balance the replicates needed for statistics and the efforts needed for sampling at each site.
Data collection	Data collection was done by the principal investigator at each site site. All NutNet sites followed standard sampling protocols, detailed in (Borer, E. T. et al. Finding generality in ecology: a model for globally distributed experiments. Methods in Ecology and Evolution 5, 63-73 (2013)). Species cover (%) was estimated visually for all species in the subplots; the total cover of living plants can exceed 100 % for multilayer canopies.
Timing and spatial scale	At most sites, cover was recorded once per year at peak biomass (peak biomass season was estimated by site PIs). At some sites with strong seasonality, cover was recorded twice per year to include a complete list of species. For those sites, the maximum cover for each species were used in the following analyses. Sampling was done in 1 m2 subplots and followed a standardized protocol at all sites. For the analyses here, we used data collected at four years and a subset at 14 years after nutrient additions began. Subplots were nested within blocks. The average pairwise distance among the three blocks within sites ranged from 23.04 to 12538.09 m, with a mean of 513.01 m and a median of 118.7 m across 54 sites that have geolocation data for each block.
Data exclusions	Exclusion criteria are pre-defined. We focused on the subset of 72 sites where 1) nutrient addition by NPK were applied for at least 4 years; and 2) each site had at least 3 blocks.
Reproducibility	To increase transparency and reproducibility, we make the R code, raw data publically available. The species cover and species richness data, site abiotic and biotic environmental data used and generated in this study have been deposited in the Figshare database and are publicly available (https://doi.org/10.6084/m9.figshare.26412295.v3). The NutNet data are publicly available on the Environmental Data Initiative (EDI) (https://portal.edirepository.org/nis/advancedSearch.jsp). The R codes used to produce results in this study have been deposited in the GitHub (https://github.com/chqq365/plant-diversity-and-biotic-homogenization.git) and archived through Zenodo (https://doi.org/10.5281/zenodo.14902812).
Randomization	Blocks within sites were randomly assigned. Treatments were randomly assigned.
Blinding	The site PIs know the location for each treatment at each site. However, all site PIs did not directly involve in data analyses. Data were analyzed using all sites together, it is difficult to know which site had which influence on the overall results.

Did the study involve field work? ☒ Yes ☐ No

Field work, collection and transport

Field conditions	Our 72 sites were distributed over 6 continents. All sites are dominated by herbaceous species. Sites cover a wide range of grassland types that range from alpine grassland, to prairie, pasture, shrub steppe, savanna and old field. Sites also cover a wide range of climate condition (e.g. latitude, longitude), and water balance (taking into account both temperature and rainfall). See below for details.
Location	See Table S1 in supplementary file of this manuscript for details of site codes, grassland types, geolocation, and experimental years used.
Access & import/export	Access to sites and data collection followed standard practices and complies with laws. No permits were needed.
Disturbance	Walkways were established to minimize trampling effects from site PIs on the sampling plots.

Reporting for specific materials, systems and methods

We require information from authors about some types of materials, experimental systems and methods used in many studies. Here, indicate whether each material, system or method listed is relevant to your study. If you are not sure if a list item applies to your research, read the appropriate section before selecting a response.

Materials & experimental systems

n/a	Involved in the study	
☒	☐	Antibodies
☒	☐	Eukaryotic cell lines
☒	☐	Palaeontology and archaeology
☒	☐	Animals and other organisms
☒	☐	Clinical data
☒	☐	Dual use research of concern
☐	☒	Plants

Methods

n/a	Involved in the study	
☒	☐	ChIP-seq
☒	☐	Flow cytometry
☒	☐	MRI-based neuroimaging

Dual use research of concern

Policy information about dual use research of concern

Hazards

Could the accidental, deliberate or reckless misuse of agents or technologies generated in the work, or the application of information presented in the manuscript, pose a threat to:

No	Yes	
☒	☐	Public health
☒	☐	National security
☒	☐	Crops and/or livestock
☒	☐	Ecosystems
☒	☐	Any other significant area

Experiments of concern

Does the work involve any of these experiments of concern:

No	Yes	
☒	☐	Demonstrate how to render a vaccine ineffective
☒	☐	Confer resistance to therapeutically useful antibiotics or antiviral agents
☒	☐	Enhance the virulence of a pathogen or render a nonpathogen virulent
☒	☐	Increase transmissibility of a pathogen
☒	☐	Alter the host range of a pathogen
☒	☐	Enable evasion of diagnostic/detection modalities
☒	☐	Enable the weaponization of a biological agent or toxin
☒	☐	Any other potentially harmful combination of experiments and agents

Plants

Seed stocks	Not applicable.
Novel plant genotypes	Not applicable.
Authentication	Not applicable.
